# Exploring the Reusability of Synthetically Contaminated Wastewater Containing Crystal Violet Dye using *Tectona grandis* Sawdust as a Very Low-Cost Adsorbent

**DOI:** 10.1038/s41598-018-26655-3

**Published:** 2018-05-29

**Authors:** Fouzia Mashkoor, Abu Nasar, Inamuddin, Abdullah M. Asiri

**Affiliations:** 1 Department of Applied Chemistry, Faculty of Engineering and Technology, Aligarh Muslim University, Aligarh, 202002 India; 2 Chemistry Department, Faculty of Science, King Abdulaziz University, Jeddah, 21589 Saudi Arabia; 3 Centre of Excellence for Advanced Materials Research, King Abdulaziz University, Jeddah, 21589 Saudi Arabia

## Abstract

Present investigation explores the possible reusability of synthetically contaminated wastewater containing crystal violet (CV) organic dye using *Tectona grandis* sawdust (TGSD) waste as a very low-cost adsorbent. The adsorbent was characterized by proximate, SEM/EDX, FTIR, and XRD analyses. Batch adsorption studies were carried under changing conditions of contact time, the initial concentration of CV, pH, TGSD dose, TGSD particle size, and temperature. The experimental data were tested using Langmuir, Freundlich and Temkin isotherm models, and the data were best followed by Langmuir one. The kinetic results were examined in the light of different models and pseudo-second-order was obtained to be best obeyed. The values of ΔH° (28.642 kJ/mol), ΔG° (-10.776 to -7.080 kJ/mol) and ΔS° (121.8 J/K/mol) in the temperature range of 293–323 K suggested the overall process to be spontaneous, endothermic and associated with an increase in randomness. On the basis of experimental results and their analyses, it has been established that TGSD is one of the most effective adsorbents among those obtained from the domestic, agricultural and industrial wastes. Thus this adsorbent can be effectively utilized to make the impure wastewater reusable.

## Introduction

Safe and clean water is essential for human health and pleasure, ecosystems and also for a booming economy^1^. Deterioration of water quality and continuous decrease in the availability of fresh water are the matters of great concerns. The presence of contaminants like heavy metals, dyes, pesticides etc. steadily degrades the quality of water and is the major reason for several diseases and damage to human health^2–6^. Among the several pollutants, dyes are the only ones which can be visible to the naked eye even at very low concentration. Depending on their nature, concentration and exposure time the effects of dyes can be acute or chronic. Dyes can cause problems such as skin irritation, respiratory diseases, mental disorder, vomiting and in many cases they may be carcinogenic and mutagenic^7–9^. Dyes are used in a number of industries like rubbers, plastics, cosmetics, textile, food, leather, pharmaceutical, wood preserving chemicals, photographic, pulp and paper, petroleum industries etc.^10–12^. There is a long list of dyes available in the market with approximately 7 × 10^5^ tonnes of annual production^13^. Crystal violet (CV) is a triarylmethane synthetic dye with deep purple hue. It has uses in paint and printing ink, veterinary medicines, etc. It also has antibacterial and antifungal properties. However, this dye has been reported to be toxic in several aspects and stays in the environment for a very long time^14^. Most of the dyes have high thermal and photostability and hard to decolourize due to their stable structure, non-biodegradability and synthetic origin^15,16^. Untreated or poorly treated effluents discharged from these industries are the reason for a serious threat to flora and fauna^17^. One of the serious environmental concerns related to dyes is that it decreases the penetration of light radiation into the water which has disparaging effects on the photosynthetic activity of aquatic life and causes a deficiency of oxygen^18,19^.

Thus, the removal of contaminants is essential to make the wastewater reusable. Different types of techniques have been developed for the treatment of water contaminants such as dyes, heavy metals, pesticides, fertilizers etc. to eliminate or reduce their hazardous impacts on the human and other living bodies. These include electrodialysis^20^, photocatalysis^21,22^, nanofiltration membranes^23,24^, electroflotation^25^, electrokinetic/electrooxidation^26^, coagulation-flocculation^12,27–29^, reverse osmosis^30^, ozonation^31,32^, colloidal manganese dioxide oxidation^4,33–36^, ion-exchange^19,37^, anaerobic-aerobic^38^, adsorption^39–42^. Out of these methods, adsorption has been observed to be most attractive one because this method is easy, effective, eco-friendly, and economical for the decontamination of dye-loaded effluents^43,44^. However, the choice of suitable adsorbent was always a challenging task. The conventional adsorbents like activated carbon and silica gel should be a primary choice but their usage is narrowed due to high cost and regeneration problems. In this context the adsorbents made from natural materials, agricultural, domestic and industrial wastes (e.g. tea waste^45^, oil palm biomass^46^, bagasse^47^, citrus limetta^48^, cucumis sativus^49^, prosopis cineraria^50^, Fe(III)/Cr(III) hydroxide^51^, titania-silica^52^, zeolite^53,54^, cupuassu shell^55^, chitosan^56–58^ etc.) offer suitable alternatives for the adsorptive treatment of dyes from wastewater.

Teak (*Tectona grandis* Linn. f.) has the extraordinary weather resistant capacity and therefore found a place as one of the most popular hardwood timber species. *Tectona grandis* is native to tropical and subtropical countries^59,60^. The sawdust of teak tree is a waste product which can be gainfully utilized as a cheap adsorbent for the elimination of dyes from the aqueous solution. The present investigation has been planned with a prime objective to find out the suitability of *Tectona grandis* sawdust (TGSD) as an adsorbent for the removal of CV from laboratory synthesized wastewater. It has also been planned to conduct the experiments on variable experimental conditions such as adsorbent dose, dye concentration, pH of the medium, adsorbate-adsorbent contact time, the particle size of adsorbent and temperature because these factors play a significant role in the optimization of the process for better feasibility.

## Materials and Methods

### Materials

CV dye of the biological stain grade, obtained from Fisher Scientific, India was used as received without further purification. The following analytical reagent grade chemicals and reagents were used in the present investigation: HCl (Fisher Scientific, India), CH_3_COOH (SD fine, India), NaOH (Merck, Germany) and KNO_3_ (CDH, India). The stock solution of CV (1000 mg/L) was made by dissolving an appropriate amount of CV in the doubly distilled deionized water (DDDW).

### Preparation and characterization of TGSD adsorbent

TGSD obtained from sawmill was cleaned comprehensively by DDDW to eliminate the dust, dirt and other surface contaminants. The washed mass was dried in sunlight under the cover of the white cotton sheet (making air gap of at least 4 cm between mass and sheet) for about 2 days and then in an oven at about 95 °C for 24 h. The dried material was crushed into the powder by a motor operated grinder and then screened through a standard sieve of 80 BSS mesh and then finally washed thoroughly with DDDW. The sieved powder (80 BSS mesh) was again dried in the oven at 105 °C for about 4 h. Thereafter the crude material mass was powderize in agate mortar pestle and separated through a set of sieves (80–150, 150–200 and >200 BSS scales) and the adsorbents of different size fractions were kept in airtight vessels.

The various components present in the TGSD adsorbent were determined by performing the proximate analysis. The Moisture content of TGSD sample was obtained by the official method of chemical analysis^61^. 2.0 g of the sample taken in a porcelain crucible was oven dried at 100 °C for 4 h. From the initial and final weight (W) of the filled and empty crucible the moisture content was calculated by using the following equation:

1 $$ \% \,{\rm{moisture}}\,{\rm{content}}=\frac{{{\rm{W}}}_{{\rm{initial}},{\rm{filled}}}-{{\rm{W}}}_{{\rm{final}},{\rm{filled}}}}{{{\rm{W}}}_{{\rm{initial}},{\rm{filled}}}{-W}_{{\rm{final}},{\rm{empty}}}}\times {\rm{100}}$$

The total ash content of the sample, based on the vaporization of water and volatile matters along with the burning of organic substances in presence of atmospheric oxygen, was measured by incinerating 2.0 g TGSD in porcelain crucible in a muffle furnace at 700 °C for 7 h. The % ash was calculated by using the following equation:

2 $$ \% \,{\rm{ash}}=\frac{{{\rm{W}}}_{{\rm{ash}}}}{{{\rm{W}}}_{{\rm{TGSD}}}}\times {\rm{100}}$$

The content of volatile matter was determined from the weights of 2.0 g of dry sample (W_dry_) and heated sample (W_heated_) at 550 °C (10 min) by using the following equation:

3 $$ \% \,{\rm{volatile}}\,{\rm{matter}}=\frac{{{\rm{W}}}_{{\rm{dry}}}-\,{{\rm{W}}}_{{\rm{heated}}}}{{{\rm{W}}}_{{\rm{dry}}}}\times {\rm{100}}$$

The percent fixed carbon was evaluated by using the following equation:

4 $$ \% \,{\rm{fixed}}\,{\rm{carbon}}=100- \% ({\rm{moisture}}+{\rm{ash}}+{\rm{volatile}}\,{\rm{matter}})$$

The morphological features of the CV unloaded and loaded TGSD were analyzed by using SEM/EDX (JEOL, JSM6510LV, Japan). FTIR spectrum of the TGSD was measured in the spectral range of 4000–400 cm^−1^ in the solid state. The adsorbent was also characterized by measuring the point of zero charge by employing the solid addition method. XRD patterns of TGSD and CV adsorbed TGSD were recorded using X-ray diffractometer (Miniflex II, Rigaku, Japan) with CuKa radiation at the scanning speed of 10°/min with 2θ angle varying in the range of 5–60°.

### Batch equilibrium studies

Batch experiments were conducted at the variable dose of TGSD, solution pH, initial concentrations of CV, the particle size of TGSD, adsorbate-adsorbent contact time and temperature. The time-dependent (3 to 180 min) measurements were conducted by adding 0.05 g of TGSD adsorbent into 25 ml of CV solution with varying initial concentration (25–150 mg/L) at room temperature. After filtering the adsorbent agitated adsorbate solution, the residual concentration of dye in the filtrate was determined by UV-VIS spectrophotometer at a pre-optimised λ_max_ (wavelength corresponding to maximum absorption) of 575 nm. The extent of dye adsorption, which is usually expressed by two parameters adsorption capacity (q) and percent removal efficiency (%R), and can be obtained by employing the following equations:

5 $${{\rm{q}}}_{{\rm{e}}}=({{\rm{C}}}_{{\rm{o}}}-{{\rm{C}}}_{{\rm{e}}})\times \frac{{\rm{V}}}{{\rm{m}}}$$

6 $$ \% {\rm{R}}=\frac{({{\rm{C}}}_{0}-{{\rm{C}}}_{{\rm{t}}})}{{{\rm{C}}}_{0}}\times 100$$

where C is a concentration of CV (mg/L), V is the solution volume (L), m is the adsorbent mass (g). The subscript o, e and t represent the terms initial, equilibrium and anytime, respectively and will be used as and when required. The adsorption capacity at any time (q_t_) can be computed by using analogous equation obtained by replacing the term C_e_ by C_t_ in equation (5).

### Adsorption isotherm

The adsorption isotherm plays a major role in the strategy of any adsorbate-adsorbent system. It gives important information on adsorption mechanism, surface properties of adsorbent, the surface affinity of adsorbate onto the absorbent. The results of the present studies have been analyzed in three commonly used isotherm models as pronounced by their inventors, namely, Langmuir^62^, Freundlich^62,63^ and Temkin^64^. The linear mathematical forms are given below in respective order:

7 $$\frac{1}{{{\rm{q}}}_{{\rm{e}}}}=\frac{1}{{{\rm{q}}}_{{\rm{m}}}{{\rm{K}}}_{{\rm{L}}}{{\rm{C}}}_{{\rm{e}}}}+\frac{1}{{{\rm{q}}}_{{\rm{m}}}}$$

8 $${{\rm{lnq}}}_{{\rm{e}}}=\frac{1}{{\rm{n}}}{{\rm{lnC}}}_{{\rm{e}}}+{{\rm{lnK}}}_{{\rm{F}}}$$

9 $${{\rm{q}}}_{{\rm{e}}}={\rm{B}}\,{{\rm{lnC}}}_{{\rm{e}}}+{\rm{B}}\,{{\rm{lnK}}}_{{\rm{T}}}$$

In the above equations, q_e_ is adsorption capacity at equilibrium, q_m_ (mg/g) is the theoretical Langmuir maximum adsorption capacity, K_L_ (L/mg) is the Langmuir adsorption constant, K_F_ (mg^1–1/n^L^1/n^/g) and n are the Freundlich constants, B (=RT/b), b (J/mol) and K_T_ (L/g) are the Temkin isotherm constants, T is the absolute temperature (K) and R is the universal gas constant (8.314 J/K/mol). Further, the important features of Langmuir isotherm may be expressed by a dimensionless quantity R_L_ $(={\frac{1}{1+{{\rm{K}}}_{{\rm{L}}}{{\rm{C}}}_{{\rm{o}}}}}_{})$, the value of which indicates the adsorption to be favourable (0–1), unfavourable (>1), linear (1) or irreversible (0).

### Adsorption kinetics

The kinetics behind the removal of CV onto the surface of TGSD was studied by monitoring the experiments as a function of time. The experimental procedure was same as performed with the batch equilibrium studies. The adsorption results were analyzed in the light of three most commonly used kinetic models namely, pseudo-first order^65,66^, pseudo-second order^67^ and intraparticle diffusion^68,69^. The linear forms of these models in the respective order may be represented as:

10 $$\mathrm{ln}({{\rm{q}}}_{{\rm{e}}}-{{\rm{q}}}_{{\rm{t}}})={{\rm{lnq}}}_{{\rm{e}}}-{{\rm{K}}}_{1}{\rm{t}}$$

11 $$\frac{{\rm{t}}}{{{\rm{q}}}_{{\rm{t}}}}=\frac{1}{{{\rm{K}}}_{2}{\,{\rm{q}}}_{{\rm{e}}}^{2}}+\frac{{\rm{t}}}{{{\rm{q}}}_{{\rm{e}}}}$$

12 $${{\rm{q}}}_{{\rm{t}}}={{\rm{K}}}_{\mathrm{id}}{{\rm{t}}}^{1/2}+{\rm{C}}$$

where K_1_(1/min), K_2_ (g/min mg), and K_id_ (mg min^−½^/g) is the first-order rate constant, second-order rate constant, and intraparticle diffusion rate constant, respectively. The values of various kinetic parameters were determined from the respective linear plots (Section 3).

### Adsorption thermodynamics

Thermodynamic factors, viz., Gibb’s free energy (∆G°), enthalpy (∆H°), and entropy (∆S°) change associated with adsorptive decontamination of CV by TGSD were calculated via the following equations:

13 $${\rm{\Delta }}{\rm{G}}^\circ =-{{\rm{RTlnK}}}_{{\rm{c}}}$$

14 $${{\rm{lnK}}}_{{\rm{c}}}=\frac{-{\rm{\Delta }}{\rm{G}}^\circ }{{\rm{RT}}}=\frac{-{\rm{\Delta }}{\rm{H}}^\circ }{{\rm{RT}}}+\frac{{\rm{\Delta }}{\rm{S}}^\circ }{{\rm{R}}}$$

where K_c_ is the equilibrium constant which is the ratio of the equilibrium concentration of dye on the adsorbent to that in the solution.

### Desorption studies

Desorption experimentations were conducted to analyze the recapture of adsorbent. In each experiment, 0.5 g of CV loaded TGSD was separately agitated with 25 ml of DDDW, 0.1 M hydrochloric acid, 0.1 M sodium chloride, 0.1 M acetic acid, 0.1 M sodium hydroxide and equilibrates for 4 h and then filtered. After filtration, the supernatant was analyzed and percent desorption (%D) was computed by employing the following relation:

15 $$ \% {\rm{D}}=\frac{{{\rm{m}}}_{{\rm{d}}}}{{{\rm{m}}}_{{\rm{a}}}}\times 100$$

where, m_a_ and m_d_ represent the concentrations of dye adsorbed and dye desorbed in mg/L, respectively.

## Results and Discussion

### Adsorbent characterization

**Figure 1:**
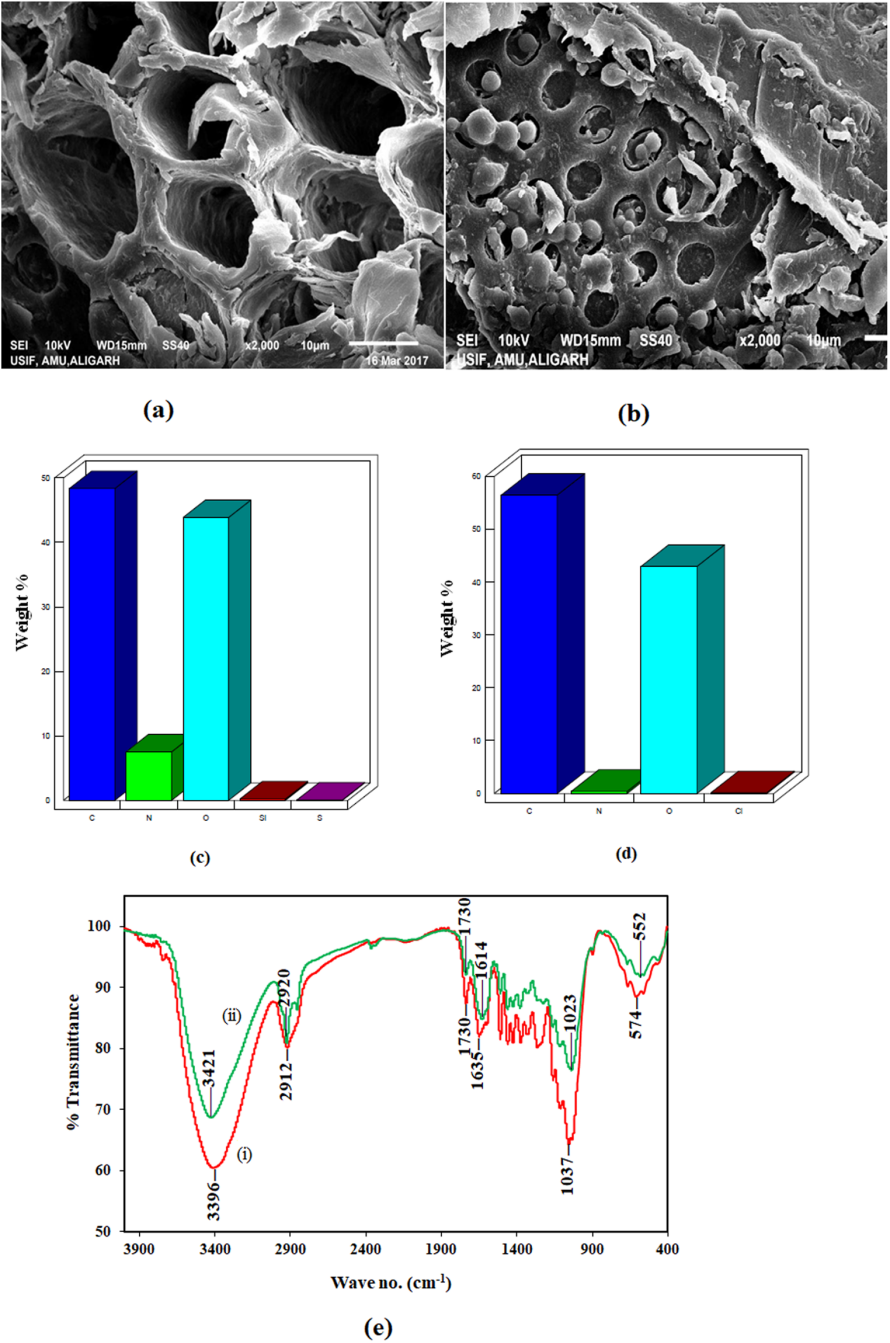
(**a**) SEM micrograph of unloaded TGSD. (**b**) SEM micrograph CV loaded TGSD. (**c**) EDX of unloaded TGSD. (**d**) EDX of CV loaded TGSD. (**e**) FTIR spectra of unloaded and CV loaded TGSD i.e. (i) before and (ii) after adsorption of CV.

The proximate analysis reveals that the TGSD adsorbent contains 5.0% moisture, 0.4% ash, 73.8% volatile matter and 20.8% fixed carbon. The SEM micrographic images of the CV dye unloaded and loaded adsorbent TGSD respectively shown in Fig. 1a,b clearly indicate that the surface of unloaded TGSD is highly irregular, porous and has cave type openings which provide a greater surface area for effective adsorption. After adsorption, the surface of the adsorbent as clearly seen in Fig. 1(b) was altered due to attachment of CV molecules. Obviously, the pores were occupied by CV after adsorption. Thus on the basis of SEM images, it can be inferred that the TGSD adsorbent has adequate morphology for dye adsorption. EDX analysis of TGSD before and after adsorption was carried out to judge the adherence of CV molecules onto the adsorbent surface. The EDX results shown in Fig. 1c,d reflect that the traces of Si and S present in unadsorbed TGSD was replaced by Cl atom of CV (C_25_H_30_ClN_3_). Weight changes in C, N and O atoms were also observed which is clearly due to adsorption of the dye molecule.

The existence of different functional groups on the surface of TGSD was determined by FTIR spectroscopy. The spectrum of TGSD adsorbent is also incorporated in Fig. 1 as Fig. 1e. This figure clearly illustrates that TGSD is enriched with a number of functional groups which are available for adsorption of dye. The strong and broad bands from 3600 to 3200 cm^−1^ attributed to the stretching vibration of a hydroxyl group and adsorbed water while the bands in the range of 3000 to 2850 cm^−1^ may correspond to C–H stretching vibration of alkane group^70^. The broadband peak with a rounded tip at 3396 cm^−1^ of TGSD spectrum is attributed to O–H stretching of an alcoholic group of adsorbent. The peak at 2912 cm^−1^ might be due to C–H stretching of an alkane. A weak band at 1730 cm^−1^ is attributed to C=O stretching non-ionic carboxyl groups (–COOH, –COOCH_3_) of carboxylic acid or its ester and a medium steep band at 1635 cm^−1^ is accounted to C=O stretching of the ionic carboxylic group (–COO^−^)^71–73^. A clear peak at 1037 cm^−1^ is due to C–N stretching of the amine group^74^ or –OH peak of carbohydrate^75^. The participation of respective functional groups during the adsorption is clear from the change in intensity and peak positions in TGSD after adsorption of CV (Fig. 1e, curve ii). It is evidently seen from the figure that there is a shift in peaks from 3396 to 3421, 2912 to 2920, 1635 to 1614 and 1037 to 1023 cm^−1^ after the adsorption. These shifts in peak indicate the involvement of respective functional groups in the adsorption of CV by TGSD. Thus alcoholic and carboxylic groups take active participation in the adsorption of cationic dye. There strong attractive forces involve between the positive centers of CV dye with the negative center of these groups.

**Figure 2:**
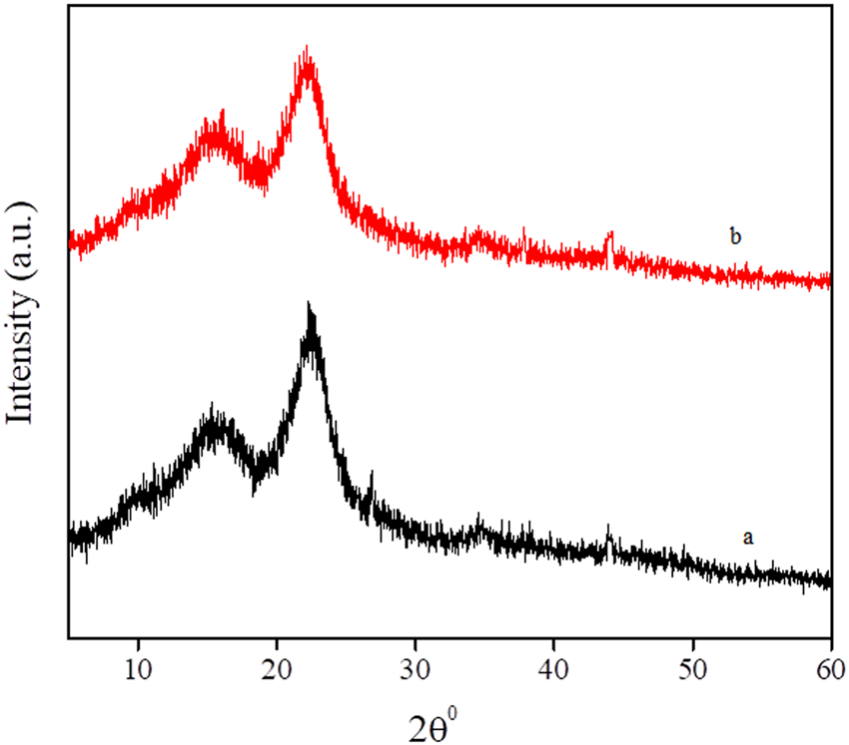
XRD Spectra (**a**) unloaded TGSD (**b**) CV loaded TGSD.

The XRD patterns in the 2θ range 5–60° depicted in Fig. 2 indicate that the present adsorbent is low crystalline material. In fact, the XRD pattern of a biomass is routinely amorphous in nature due to the presence of hemicellulose and lignin^73^. However, two broad peaks observed at the 2θ values of 15.3 and 22.3° are due to the crystalline region of cellulose in TGSD. The broad peaks at around 16° and 22° were also reported by other investigators on different woods such as rubber wood sawdust^76^, pinewood^77^ and *pinus elliotii* plantation wood^78^. Further Fig. 2 clearly indicates that there is no significant change in crystallinity of the TGSD after the adsorption of CV dye. Thus the crystallinity of TGSD is not affected by the adsorption of dye molecules. This observation rules out the possibility of secondary doping^79^.

### Effect of contact time and initial concentration of dye

**Figure 3:**
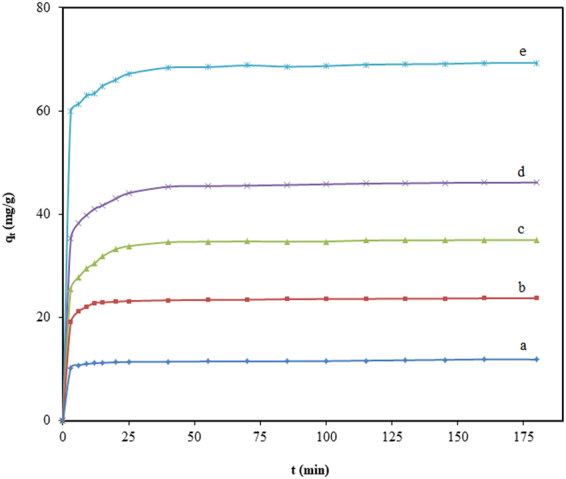
Effect of contact time on adsorption of CV onto TGSD at different initial dye concentrations: (**a**) 25 mg/L, (**b**) 50 mg/L, (**c**) 75 mg/L, (**d**) 100 mg/L, (**e**) 150 mg/L (experimental conditions: C_o_ = 50 mg/L, temperature = 298 K, adsorbent dose = 2 g/L and pH = 7.5).

**Figure 4:**
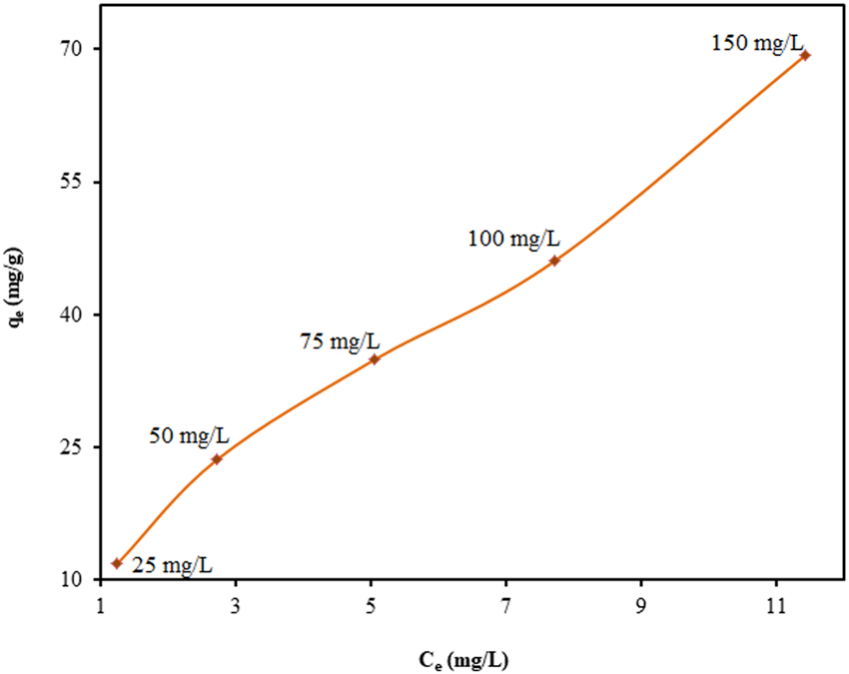
Effect of initial dye concentration on adsorption of CV onto TGSD (experimental conditions: temperature = 298 K, contact time = 180 min, adsorbent dose = 2 g/L and pH = 7.5).

Figure 3 shows the influence of TGSD/CV agitation time on adsorption capacity at the different initial concentrations of dye. It can be realized from the figure that removal efficiency (%) increases with contact time and attained equilibrium in 180 min. After which there is no considerable change in adsorption capacity. However, during the initial 25 min, the rapid increase in adsorption capacity is observed which may be due to a greater number of vacant sites available on the boundary layer of TGSD. The slow increase at the later stage was due to a continuous reduction in the vacant sites. Further adsorption of CV became difficult due to the repulsion of solute between solid and bulk phase. After the initial stage adsorption (i.e. after about 25 min.), the surface pores get almost saturated with CV. Therefore, at the later stage of adsorption, the molecules of CV have to traverse farther and deeper into the micropores of the adsorbent which encounters much larger resistance. Figure 4 also suggests that the adsorption capacity increases with the increase in initial dye concentration at any time. This is due to the fact that at the higher initial dye concentration provides a more driving force which accelerates the mass transfer of adsorbate to the adsorbent. Thus the initial dye concentration plays a significant role in the adsorption capacity in any adsorbate-adsorbent system.

### Effect of adsorbent dose

**Figure 5:**
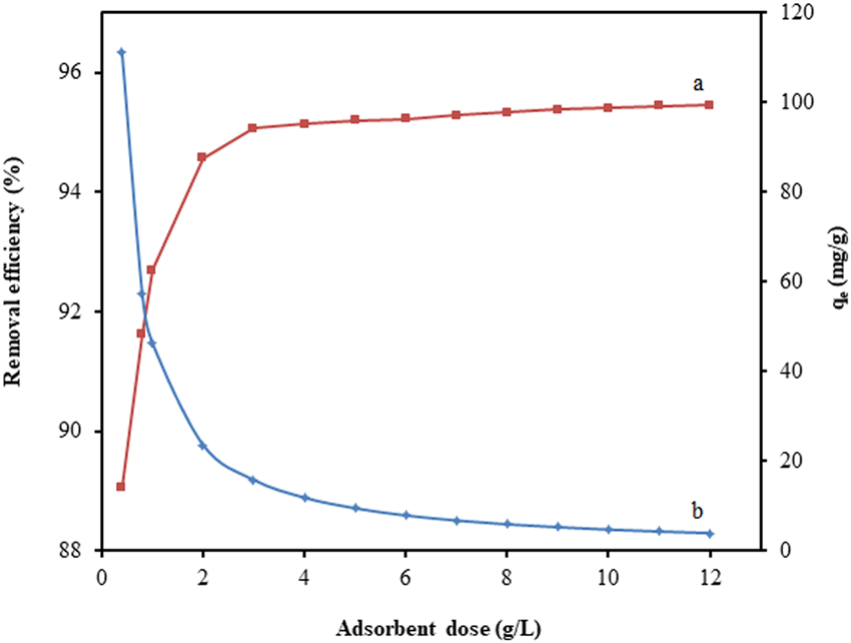
Effect of adsorbent dosage on (**a**) removal efficiency and (**b**) adsorption capacity (experimental conditions: C_o_ = 50 mg/L, temperature = 298 K, contact time = 180 min and pH = 7.5).

The adsorption of CV onto TGSD was studied by changing the adsorbent dose ranging from 0.4 to 12 g/L. The values of adsorption capacity and removal efficiency have been plotted against TGSD dose at the typically chosen experimental conditions of C_o_ = 50 mg/L, temperature = 298 K, contact time = 180 min and pH = 7.5 in Fig. 5. It can be noticeably realized that the removal efficiency increases significantly from 89 to 95% with increased in adsorbent dose from 0.4 to 2.0 g/L and thereafter the increase is relatively slow or insignificant. The increase in removal efficiency with increasing adsorbent dose is due to increase in the overall surface area of the adsorbent and accordingly availability of more binding sites for adsorption. However, a reverse trend of adsorption capacity has been observed. Figure 5 clearly indicates that the adsorption capacity decreases drastically from 111.0 to 3.9 mg/g with the increase in adsorbent dose from 0.4 to 12.0 g/L. The continuous decrease in adsorption capacity with an increase in TGSD dose is due to the cohesive interaction of adsorbent particles like aggregation or agglomeration which brought about the decrease of the effective surface area per unit weight (g) of the adsorbent and increase in diffusion path length. In view of both positive and negative aspects of increasing adsorbent dose towards removal efficiency and adsorption capacity, a judicious value of 2.0 g/L of the adsorbent dose has been selected for further studies. This adsorbent dose corresponds to a removal efficiency of 94.6% and adsorption capacity of 23.74 mg/g at the initial dye concentration of 50 mg/L.

### Effect of pH

**Figure 6:**
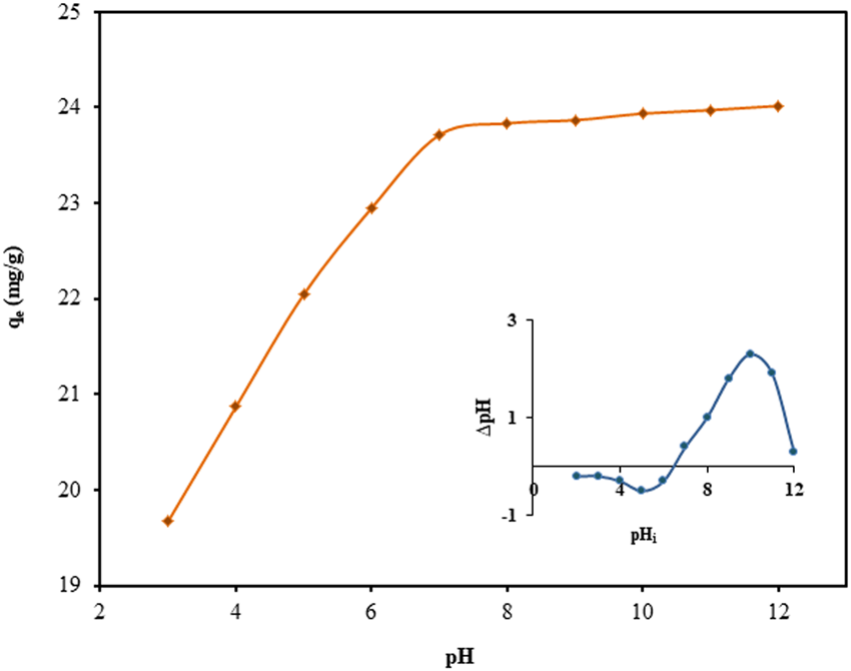
Effect of initial pH on adsorption capacity (inset – determination of the point of zero charge) (experimental conditions: C_o_ = 50 mg/L, temperature = 298 K, contact time = 180 min and adsorbent dose = 2 g/L).

The pH of the medium is a significant parameter for adsorption of dye, as it controls the surface charge of the adsorbent as well as the degree of ionization of the solute. The effect of pH was studied by changing the pH of CV solution from 3 to 12. The effect of pH on the adsorption capacity at the experimental conditions of C_o_ = 50 mg/L, temperature = 298 K, contact time = 180 min and adsorbent dose = 2.0 g/L, has been plotted in Fig. 6. This figure clearly indicates that as the pH of the medium is increased from 3 to 7, the value of q_e_ has been observed to increase drastically, due to increasing electrostatic attraction between cationic CV dye and TGSD surface. In this context, it is relevant to comment that with the increase of pH there is an increase in negative charge of the TGSD surface which causes deprotonating of the functional group present on the adsorbent. Hence, these deprotonated functional groups serve as the binding sites for the cationic CV and cause better adsorption. The lower adsorption of dye in acidic medium is apparently due to electrostatic repulsion of the positively charged adsorbent surface with cationic CV dye. The profound influence of pH on the adsorption can be explained in a better way by an adsorbent characteristic known as the point of zero charge (pH_pzc_).

The pH_pzc_ of TGSD adsorbent, measured by employing solid-addition method^80^, was found to be 6.5 (Fig. 6 – inset). This indicates that at pH < 6.5 the surface has a positive charge due to the protonation, whereas, at pH > 6.5, the adsorbent surface acquires negative charged due to deprotonating in the excess of hydroxyl ions^81^. Thus a value of 6.5 for pH_pzc_ further indicates that the TGSD adsorbent favourably adsorbed any cationic dye at pH of the medium greater than this value. In view of the above facts, an optimum pH value of 7.5 was chosen for further investigation.

### Effect of particle size

Experiments on the adsorptive decontamination CV by TGSD adsorbent were conducted by taking three different size fractions of TGSD powder (80–150, 150–200 and >200 BSS mesh) and the outcomes are incorporated in Table 1. This table demonstrates that as the particle size is decreased from 80–150 BSS to >200 BSS range, the removal efficiency increases from 95.1 to 99.6%. The increase in removal efficiency with decreasing particle size is due to increase in the effective surface area of adsorbent in the same order. However, due to the problems in handling the smaller particle size, particles of 80–150 BSS size fraction were selected throughout the further experiments.

**Table 1:** Effect of particle size on removal efficiency (experimental conditions: C_o_ = 50 mg/L, temperature = 298 K, contact time = 180 min, adsorbent dose = 2.0 g/L, pH = 7.5).

Particle size (BSS mesh)	% R
80–150	95.1
150–200	98.8
>200	99.6

### Adsorption isotherm

**Figure 7:**
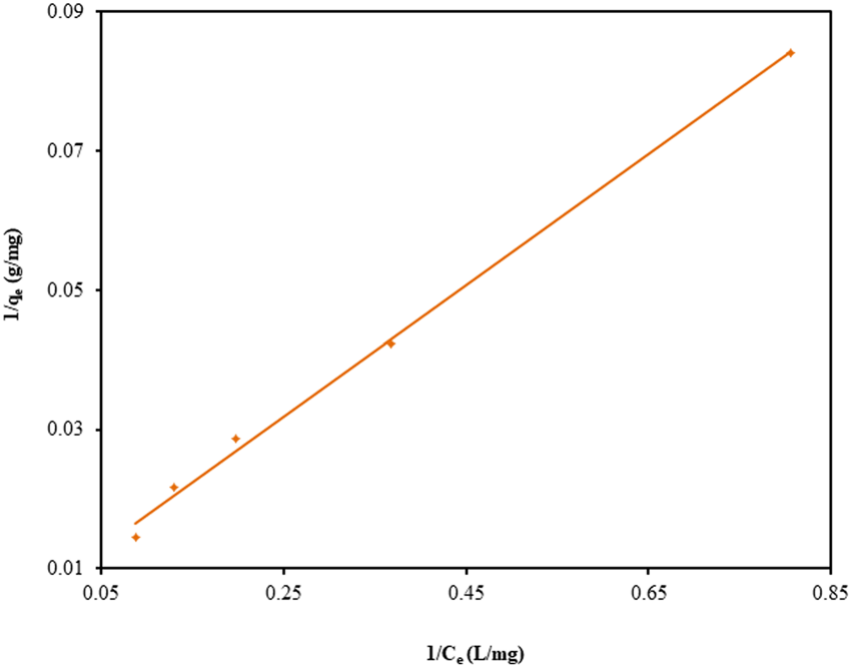
Langmuir adsorption isotherm for the adsorption of CV onto TGSD (experimental conditions: temperature = 298 K, contact time = 180 min, adsorbent dose = 2 g/L, and pH = 7.5).

The isotherm modelling is very significant for the design of any adsorbate-adsorbent system. The present equilibrium results have been fitted to three different isotherm models, namely, Langmuir, Freundlich, and Temkin. The different isotherm parameters based on these models for the adsorption of CV by TGSD have been summarized in Table 2. On comparing the R^2^ values so obtained for these models, it has been established that the Langmuir model is best-fitted one. Table 2 openly indicates that our isotherm results are best represented by Langmuir model. The linear form of Langmuir isotherm (equation (7)) plotted in Fig. 7 (experimental conditions: temperature = 298 K, contact time = 180 min, adsorbent dose = 2.0 g/L and pH = 7.5) clearly indicated the excellent validity of this isotherm in CV-TGSD adsorbate-adsorbent system. Thus the adsorption of CV onto TGSD is associated with the monolayer coverage and the adsorbent surface possesses a finite number of adsorption sites with constant energy of adsorption. The validity of Langmuir isotherm also confirms that no further adsorption is possible once the adsorption occurs at the active sites of adsorbent. The favorability of the adsorption has been confirmed by evaluating the R_L_ value. A value of 0.345 for this parameter confirms that the adsorption of CV on TGSD is favourable. Further, a value of 0.7696 for 1/n as obtained from Freundlich isotherm clearly indicate a normal Langmuir isotherm and ruled out the possibility of cooperative adsorption^82^. Literature survey reveals that adsorptive decontamination of CV by other adsorbents such as agaricus bisporous^83^, ananas comosus^84^, breadfruit skin^85^, calotropis procera peel^86^, cucumis sativus and cucumis sativus/H_2_SO_4_-modified^87^, eggshell^88^, formosa papaya seed powder^89^, grapefruit peel^71^, jackfruit leaf powder^90^, jalshakti polymer^91^, jute fibre carbon^92^, natural clay mineral^93^, rice husk/NaOH-modified^94^, sawdust^95^, syzygium cumini leaves^96^, tea dust^45^, tomato plant root^97^, water hyacinth^98^ has also been observed to follow Langmuir isotherm. The values of isotherm constants of this model as reported for different adsorbents are listed in Table 3. This table clearly indicates that there is a large variation in the Langmuir adsorption capacity (q_m_) obtained by different investigators. In fact, the maximum monolayer adsorption capacity is strongly dependent on the experimental conditions. For example, the effect of adsorbent dose on the adsorption capacity is highly remarkable (Fig. 5). From Table 3 it may safely be inferred that the TGSD is one of the most prominent and effective adsorbents for the decolorization of wastewater.

**Table 2:** Langmuir, Freundlich, Temkin constant for the adsorption of CV onto TGSD (experimental conditions: temperature = 298 K, contact time = 180 min, adsorbent dose = 2 g/L, pH = 7.5).

Isotherm models	Parameters	Values	R^2^
Langmuir	q_m_ (mg/g)	131.58	0.998
	K_L_ (L/mg)	0.038	
	R_L_	0.345	
Freundlich	n	1.2994	0.989
	K_F_ (mg^1–1/n^L^1/n^/g)	10.31	
Temkin	B	22.784	0.961
	K_T_ (L/g)	0.565

**Table 3:** Langmuir isotherm values for the adsorption of CV onto the different adsorbent.

Adsorbent	Dose (g/L)	K_L_ (L/mg)	q_m_ (mg/g)	R^2^
Agaricus bisporous^83^	4	0.15	82.98	1.0
Ananas comosus^84^	2	0.861	78.227	0.999
Breadfruit skin^85^	—	7.35 × 10^−5^	145.8	0.9922
Calotropis procera peel^86^	10	0.1139	4.14	—
Cucumis sativus^87^	4	0.076	33.22	0.995
Cucumis sativus/H_2_SO_4_^87^	4	0.216	35.33	0.989
Eggshell^88^	30	1.341	70.032	0.997
Formosa papaya seed powder^89^	12	0.0015	85.99	0.986
Grapefruit peel^71^	1	0.131	249.68	0.994
Jackfruit leaf powder^90^	10	2.786	43.39	0.999
Jalshakti polymer^91^	0.8	2.22	12.9	0.98
Jute fiber carbon^92^	1	0.227	27.999	0.956
Natural clay mineral^93^	0.5	0.0162	330	0.96
Rice husk/NaOH^94^	10	5.632	44.87	0.992
Sawdust^95^	—	0.68	37.83	0.99
Syzygium cumini leaves^96^	2	3.739	38.750	0.9944
Tea dust^45^	10	0.032	175.4	0.98
Tomato plant root^97^	40	0.02	94.34	0.9918
Water hyacinth^98^	1	0.688	322.58	0.964
*Tectona grandis* Sawdust (Present study)	2	0.038	131.58	0.998

### Adsorption kinetics

**Figure 8:**
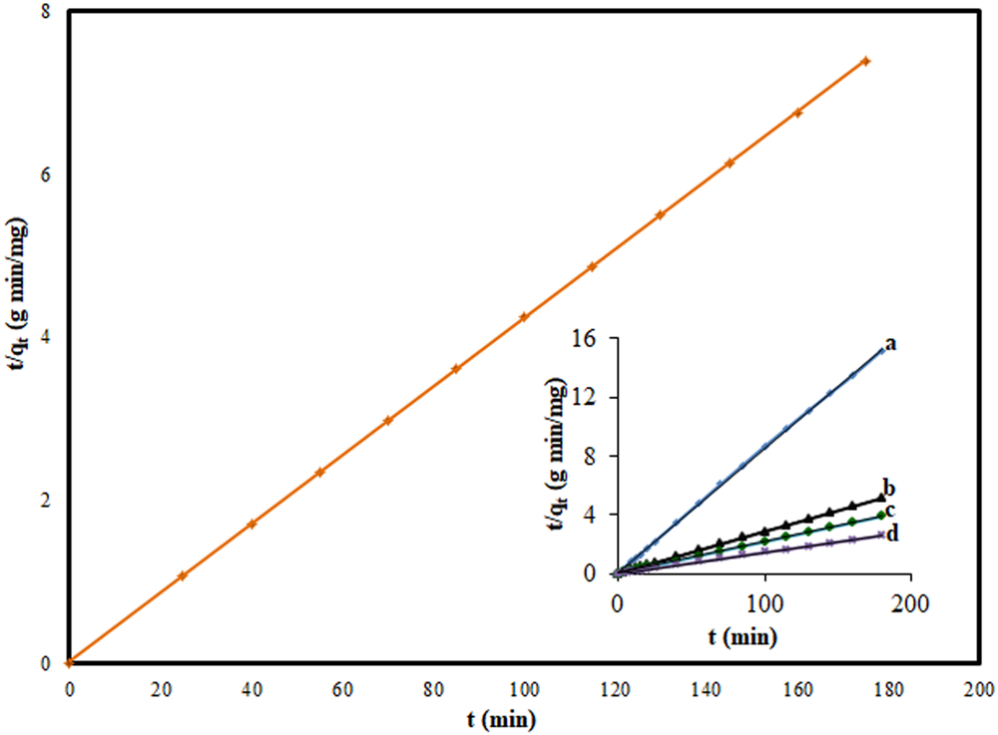
Pseudo-second order kinetic plot the adsorption of CV onto TGSD at concentration of 50 mg/L (R^2^ = 1) (inset – at different concentrations: (**a**) 25 mg/L (R^2^ = 0.9998) (**b**) 75 mg/L (R^2^ = 0.9999) (**c**) 100 mg/L (R^2^ = 1) (**d**) 150 mg/L (R^2^ = 1) (experimental conditions: C_o_ = 50 mg/L, temperature = 298 K, adsorbent dose = 2 g/L, and pH = 7.5).

The kinetic results so obtained in the present investigation have been f itted on the pseudo-first-order, pseudo-second-order and intraparticle diffusion models and the relevant parameters associated with these models are incorporated in Table 4. The values of correlation coefficient R^2^ linked with different kinetic models clearly tell that the experimental results can be best represented by the pseudo-second-order kinetic model. The plot of experimental results (t/q_t_ against t) for pseudo-second-order kinetics for different initial dye concentrations shown in Fig. 8 lead to the straight line with the excellent correlation coefficient values ranging from 0.9998 to 1.0. The calculated values of pseudo-second-order kinetic parameters for the initial dye concentration of 50 mg/L have been incorporated in Table 4. This table indicates that the experimental value of adsorption capacity (23.74 mg/g) is in a very close agreement with the value of 23.81 mg/g as calculated by employing pseudo-second-order equation. This again confirms that the kinetics of CV-TGSD system obeys pseudo-second-order model.

**Table 4:** Kinetic parameters for the adsorption of CV onto TGSD (experimental conditions: C_o_ = 50 mg/L, temperature = 298 K, adsorbent dose = 2 g/L, pH = 7.5).

Kinetic models	Parameters	Values	R^2^
Pseudo-first-order	K_1_ (min^−1^)	0.0246	0.9009
	q_e,exp_ (mg/g)	23.74	
	q_e, cal_ (mg/g)	1.76	
Pseudo-second-order	K_2_ (g /min mg)	0.0565	1.000
	q_e exp_ (mg/g)	23.74	
	q_e cal_ (mg/g)	23.81	
Intraparticle diffusion	k_id_ (mg min^−½^/g)	0.2237	0.5261
	C (mg/g)	21.277

This model has also been reported to be followed by the adsorption of CV onto other similar adsorbents, namely, agaricus bisporous^83^, alligator weed^99^, ananas comosus^84^, breadfruit skin^85^, cucumis sativus and cucumis sativus/H_2_SO_4_-modified^87^, eggshell^88^, formosa papaya seed powder^89^, grapefruit peel^71^, jackfruit leaf powder^90^, jute fibre carbon^92^, laminaria japonica^99^, rice bran^99^, rice husk/NaOH-modified^94^, sawdust^95^, syzygium cumini leaves^96^, tea dust^45^, water hyacinth^98^ and wheat bran^99^. The different kinetic parameters obtained by these investigators have been compared with those of present study in Table 5. This table clearly indicates that our value of 0.0565 g/min mg for the Pseudo-second-order rate constant (K_2_) falls under the comparable range of literature values (0.0017–0.15 g/min mg).

**Table 5:** Pseudo-second-order kinetic parameters for adsorption of CV onto different adsorbents.

Adsorbent	K_2_ (g/min mg)	q_cal_ (mg/g)	R^2^
Agaricus bisporous^83^	0.15	19.18	1.000
Alligator weed^99^	2.6 × 10^2^	14.5	0.99
Ananas comosus^84^	0.00625	74.262	0.992
breadfruit skin^85^	0.061	118.31	0.9056
Cucumis sativus^87^	0.04	12.077	0.9999
Cucumis sativus/H_2_SO_4_^87^	0.019	12.091	0.9956
Eggshell^88^	0.00725	68.056	0.9996
Formosa papaya seed powder^89^	0.078	1.890	0.993
Grapefruit peel^71^	0.005	24.31	0.992
Jackfruit leaf powder^90^	0.0193	39.45	0.999
Jute fiber carbon^92^	0.004	19.164	0.998
Laminaria japonica^99^	0.7 × 10^2^	16.1	0.99
Rice bran^99^	1.9 × 10^2^	15.2	0.99
Rice husk/NaOH^94^	0.00234	42.053	0.998
Sawdust^95^	0.0017	28.74	0.998
Syzygium cumini leaves^96^	0.014	51.020	0.9368
Tea dust^45^	0.002	45.46	0.9999
Water hyacinth^98^	0.05	434.78	1
Wheat bran^99^	0.8 × 10^2^	15.8	0.99
*Tectona grandis* Sawdust (Present study)	0.0565	23.81	1.000

### Adsorption thermodynamics

**Figure 9:**
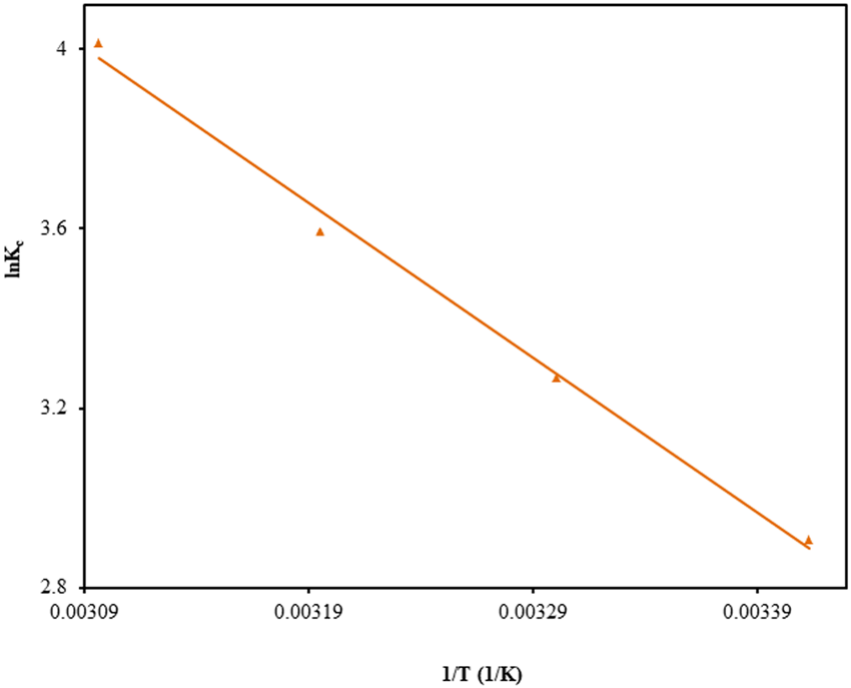
Plot of ln K_c_ vs 1/T for the adsorption of CV onto TGSD adsorbent (experimental conditions: C_o_ = 50 mg/L, contact time = 180 min, adsorbent dose = 2 g/L, and pH = 7.5).

The values of ∆G° calculated by using equation (13) and other thermodynamic parameters, namely, ∆H^o^ and ∆S^o^ as generated from ln K_c_ vs 1/T plot shown in Fig. 9 are presented in Table 6. The adsorption process has been observed to be accompanied by the decrease in free energy throughout the entire temperature range. This reveals that the adsorption of CV by TGSD is feasible. This table shows that the negative values of free energy change increase with the increase of temperature. This shows that the spontaneity of the adsorption increases with the rise of temperature. The positive value enthalpy change specifies that the adsorption is endothermic and accompanying with the absorption of heat from the surroundings. Hence the feasibility of the adsorption depends solely on the increase of entropy during adsorption CV by TGSD. A positive value of 121.8 J/K mol shows that there is an increase in disorder at the adsorbate/adsorbent interface. The enhancement in entropy is due to displacement of the coordinated water molecule by the dye molecules which resulted in a gain of more translational entropy than the lost by dye molecules^100^. This increase in disorder or randomness provides the driving force for the adsorption. Literature survey reveals that increase in randomness has also been observed for removal of CV by other adsorbents, such as alligator weed^99^, bottom ash^16^, deoiled soya^16^, lignified elephant grass complexed isolate^101^, laminaria japonica^99^, natural clay mineral^93^, rice bran^99^, terminalia arjuna sawdust^102^, tomato plant root^97^ water hyacinth^98^, wheat bran^99^, etc.

**Table 6:** Thermodynamic parameters for the adsorption of CV onto TGSD (experimental conditions: C_o_ = 50 mg/L, contact time = 180 min, adsorbent dose = 2.0 g/L, pH = 7.5).

Temperature (K)	−∆G_o_ (kJ/mol)	∆H_o_ (kJ/mol)	∆S_o_ (J/K mol)
293	7.080		
303	8.230	28.642	121.8
313	9.352		
323	10.776		

### Adsorption mechanism

The FTIR analysis has indicated the presence of polar hydroxyl, carboxyl and amine groups in TGSD. It has been pointed out that the adsorption of cationic dyes is mainly due to –OH and –COO^−^ functional groups^72^. Thus the adsorption of CV onto TGSD might be owing to the strong electrostatic attraction of these groups with the charged centers of cationic CV (CV^+^Cl^−^) molecules. Further at higher pH (i.e. > pH_pzc_), the OH^−^ ions accumulate on the surface of TGSD and accordingly the surface acquires a negative charge. In the present investigation, the adsorption of CV by TGSD was found to be strongly pH dependent (Fig. 6) and the favourable pH of the solution for CV adsorption was ranging from 7 to 12. Thus the adsorption capacity becomes significant at higher pH due to electrostatic attraction between negatively charge adsorbent surface and cationic CV molecules. The attachment of CV on TGSD occurred due to electrostatic attraction of cationic dye with the negatively charged adsorbent containing the above mentioned functional groups (–OH and –COO^−^). The adsorption of CV molecules on TGSD adsorbent may be mechanistically represented as:

16 $${\rm{T}}{\rm{G}}{\rm{S}}{\rm{D}}-{\rm{O}}{\rm{H}}\begin{array}{c}\mathop{\to }\limits^{{{\rm{O}}{\rm{H}}}^{-}}\\ {\textstyle \text{-}}{{\rm{H}}}_{2}{\rm{O}}\end{array}{\rm{T}}{\rm{G}}{\rm{S}}{\rm{D}}-{{\rm{O}}}^{-}\,\begin{array}{c}\mathop{\to }\limits^{{{\rm{C}}{\rm{V}}}^{+}{{\rm{C}}{\rm{l}}}^{-}}\\ {\textstyle \text{-}}{{\rm{C}}{\rm{l}}}^{-}\end{array}\,{\rm{T}}{\rm{G}}{\rm{S}}{\rm{D}}-{\rm{O}}-{\rm{C}}{\rm{V}}$$

17 $${\rm{T}}{\rm{G}}{\rm{S}}{\rm{D}}-{{\rm{C}}{\rm{O}}{\rm{O}}}^{-}+{{\rm{C}}{\rm{V}}}^{+}{{\rm{C}}{\rm{l}}}^{-}\,\mathop{\longrightarrow }\limits_{{\textstyle \text{-}}{{\rm{C}}{\rm{l}}}^{-}}\,{\rm{T}}{\rm{G}}{\rm{S}}{\rm{D}}-{\rm{C}}{\rm{O}}{\rm{O}}-{\rm{C}}{\rm{V}}$$

**Figure 10:**
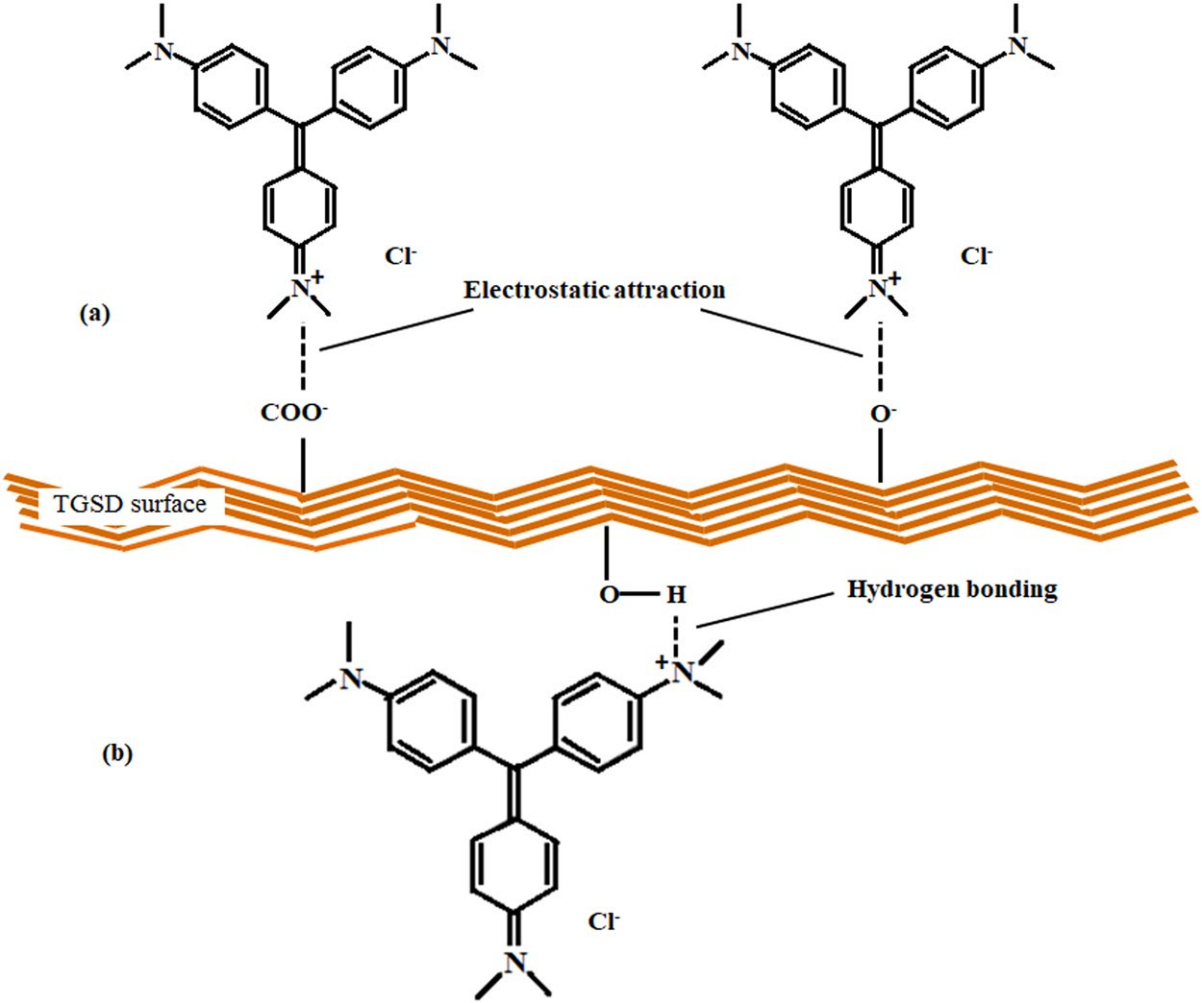
Proposed plausible interaction of crystal violet dye on TGSD Adsorbent.

In the above possible reactions, the cationic CV dye (C_25_N_3_H_30_Cl) is written as CV^+^Cl^−^. The suggested mechanism is reinforced by the experimental observation of the poor adsorption at lower pH. At the lower pH i.e. under acidic condition, the generation of Cl^−^ ions would be suppressed resulting the overall suppress of adsorption due to carboxylate ion of the adsorbent. This proposed mechanism based on the electrostatic force between positive centers of CV and –OH/–COO^−^ groups of adsorbent has been graphically presented in Fig. 10(a). The similar mechanism based on the electrostatic attraction between –COO^−^ of grapefruit peel and positive N site of CV dye has already been proposed^71^.

Furthermore, in addition to the involvement of the attractive forces between the cationic site of CV and –OH/–COO^−^ functional groups of TGSD, the involvement of the possible hydrogen bonds between alcoholic –OH group of adsorbent and amine group of adsorbate CV is quite justified. The possible formation of hydrogen bonding is illustrated in Fig. 10(b). In this context, it is relevant to mention here that the adsorption of CV on the surface of NaOH modified rice husk was suggested by the involvement of hydrogen bonding between the –OH groups on the rice husk surface and the nitrogen atoms of CV^94^ as similar as proposed in Fig. 10(b).

The probability of intraparticle pore diffusion of CV can be judged by intraparticle diffusion kinetic model. It has been suggested that the q_t_ vs t^½^ plot should linear and pass through the origin if the intraparticle diffusion is the only rate-limiting step^103^. But in the present case, a positive intercept of 21.277 mg/g (Table 4) was observed which showed that the dye removal kinetics may be governed by both film diffusion and intraparticle diffusion together^103,104^.

Thus on the basis of the above findings, the adsorption mechanism of CV onto TGSD can safely to be assumed to be involved in the following consecutive steps: (i) movement of CV molecules from the solution bulk to the surface of the TGSD, (ii) diffusion of CV molecules through the boundary layer to the surface of TGSD adsorbent, and (iii) adsorption of CV on the surface of TGSD by the (a) electrostatic attractive force between polar groups (–OH and –COO^−^) of TGSD and charged centers of CV (CV^+^Cl^−^) molecules and (b) strong hydrogen bonding between the hydroxyl group of TGSD and amine group of CV.

### Desorption

The possible regeneration of used TGSD adsorbent was explored by conducting desorption studies with different desorbing agents (0.1 M HCl, 0.1 M CH_3_COOH, 0.1 M NaCl, 0.1 M NaOH and DDDW) and the results are summarised in Table 7. The maximum desorption (33.37%) of CV was observed with hydrochloric acid. Thus in order to increase the desorption i.e. better utility adsorbent and economic feasibility of the process, further work is needed in this direction.

**Table 7:** Percentage desorption of CV from CV loaded TGSD.

Desorbing agent	% Desorption
HCL	33.37
CH_3_COOH	18.94
NaOH	5.86
NaCl	3.62
DDW	1.40

## Conclusions

The present investigation reflects that *Tectona grandis* sawdust (TGSD) is an efficient adsorbent for the decontamination of crystal violet (CV) dye from wastewater. It was observed that the adsorption capacity was influenced by different investigational variables like contact time, adsorbent dose, pH, the concentration of dye and temperature. The adsorption capacity was observed to be increased with increasing pH of the medium up to a value of 7 and thereafter it remains almost stagnant on a further increase of pH. Equilibrium time for adsorption of CV onto TGSD was found to be 180 min. Kinetic study revealed that adsorption was best described by pseudo-second-order kinetics. Adsorption equilibrium data were found to be best fitted by Langmuir isotherm model suggesting that the adsorption occurred in a monolayer manner on the homogeneous adsorbent surface having identical sites. The maximum equilibrium adsorption capacity was observed to be 131.58 mg/g which suggested that the present adsorbent is one of the most promising among the adsorbents of the similar category. The high value of equilibrium adsorption capacity clearly suggests that the present method can be successfully exploited to make impure wastewater to be reusable. The thermodynamic study showed that the decontamination of CV by TGSD was spontaneous, endothermic and associated with an increase in entropy. The desorption of TGSD from CV loaded TGSD for reuse was observed to be highest with 0.1 M HCl. The exhaustive studies indicated that the TGSD is one of the most prominent and effective adsorbents, for the decontamination of CV polluted wastewater, among the other adsorbents of similar category.
